# CircRNA_101491 regulated the radiation sensitivity of esophageal squamous cell carcinomas via sponging miR-125a-5p

**DOI:** 10.1186/s13014-024-02478-7

**Published:** 2024-06-26

**Authors:** Chen Lin, Xianfeng Huang, Yuchen Qian, Jiayi Li, Youdi He, Huafang Su

**Affiliations:** 1 Department of Radiation Oncology, The First Affiliated Hospital of Wenzhou Medical University, Wenzhou, 325000 China; 2 Zhejiang Key Laboratory of Intelligent Cancer Biomarker Discovery and Translation, First Affiliated Hospital of Wenzhou Medical University, Wenzhou, 325035 China

**Keywords:** CircRNA_101491, miR-125a-5p, EMT, ESCC, Radiosensitivity

## Abstract

**Background:**

At present, it has been found that many patients have acquired resistance to radiotherapy, which greatly reduces the effect of radiotherapy and further affects the prognosis. CircRNAs is involved in the regulation of radiosensitivity of many kinds of tumor cells. Therefore, the main purpose of this study is to explore the regulatory effect of CircRNA_101491 on radiosensitivity of ESCC and its related mechanism.

**Methods:**

We established ESCC radiation-resistant cell line (KYSE150R cell) by gradient dose method, and tested the difference of KYSE150 between KYSE150R cell and parent cell in vitro. Then, after knocking down the expression of CircRNA_101491, a series of in vitro experiments were conducted to verify the effects of CircRNA_101491 on the phenotype and radiosensitivity of KYSE150R cells, and further analyzed the related regulatory mechanism. In addition, we also used the model of transplanted tumor in nude mice to investigate the effect of CircRNA_101491 on the radiosensitivity of ESCC in vivo.

**Results:**

According to a series of in vitro experiments, we confirmed that KYSE150R cells lost the epithelial phenotype and obtained interstitial cell-like phenotype, and found that CircRNA_101491 was highly expressed in KYSE150R cells. In addition, we found that knocking down the expression of CircRNA_101491 will lift the inhibition of miR-125a-5p, and then reverse the process of EMT, accelerate the process of apoptosis, thus play a role in radiosensitization. The in vivo experiment of transplanted tumor in nude mice also showed that knocking down the expression of CircRNA_101491 could enhance the radiosensitivity of ESCC.

**Conclusion:**

In conclusion, we confirmed that interfering with the expression of CircRNA_101491 can relieve the inhibition of miR-125a-5p, thus reverse the process of interstitial phenotype, accelerate the process of apoptosis, and enhance the radiosensitivity of ESCC.

**Supplementary Information:**

The online version contains supplementary material available at 10.1186/s13014-024-02478-7.

## Introduction

Esophageal squamous cell carcinoma (ESCC) [1–3] is the most common malignant tumor of the upper digestive tract in China. Its incidence is as high as 456,000, of which about 400,000 died. In addition, the incidence of ESCC in men is three times higher than that in women [4]. More than 40% of patients with esophageal cancer still have recurrence after operation. Simultaneous radio-chemotherapy is the standard treatment for advanced esophageal cancer, but 57.4% of the patients have local recurrence, 38.3% of the patients have distant metastasis, and the 5-year survival rate is less than 20%[5]. The occurrence of acquired radiotherapy resistance is an important cause of treatment failure and recurrence of esophageal cancer.

Existing studies have shown that hypoxia [6], DNA damage repair [7], oncogene or tumor suppressor gene mutations [8] or changes in signal transduction pathway [9] in the microenvironment are important reasons for tumor radiotherapy resistance. However, the mechanism of radiotherapy resistance of esophageal cancer is not clear. Epithelial-mesenchymal transition (EMT) [10] is still a hot topic in the field of tumor, which is closely related to the drug resistance of tumor cells. At the same time, EMT is the key link of tumor acquired radiotherapy resistance. EMT can make tumor cells acquire the ability of anti-apoptosis, and then produce radiotherapy tolerance [11].

CircularRNAs (circRNAs) [12–14] is a covalently linked non-coding RNA, which is highly conserved in different tissues, has a stable ring structure and high tolerance to nuclease, plays an important role in transcriptional and post-transcriptional gene expression, and has unique advantages as a competitive endogenous RNA (Cerna). It was found [15] that the expression of hsa-circ002059 in gastric cancer was significantly down-regulated, which was closely related to distal metastasis, lymph node metastasis, sex and age of patients. In addition, some studies [16] have found that the expression of Hsa_circ_100855 is up-regulated and hsa-circ104912 is down-regulated in laryngeal carcinoma, and its expression level is related to tumor stage, differentiation and lymph node metastasis, which is expected to become a tumor marker of laryngeal carcinoma. Wei Zhao et al. [17]. determined the carcinogenic effect of circUHRF1 on the occurrence and development of OSCC tumor by circUHRF1/miR-526b-5p/c-Myc/TGF- β 1/ESRP1 feedback loop. In addition, studies [18, 19] have found that 6-methyladenosine (m [5]A) modification is the most common modification in eukaryotic RNA, so m [6]A can also modify circRNA to affect the biological activity. At the same time, circRNAs is abnormally expressed in cervical cancer cells [20], hepatic stellate cells [21] and esophageal cancer cells [22], which plays an important role in regulating the radiosensitivity of these cancer cells. However, few studies have focused on the regulatory effect of circRNAs on the radiosensitivity of ESCC. On the basis of our previous work [23], the expression profiles of radioresistant KYSE150R and radiosensitive KYSE150 cells were detected by AgilenthumancircRNA microarray. It was found that compared with KYSE150 cells, there were 74 circRNAs with more than 2-fold difference in radiation-resistant KYSE150R group, including 57 up-regulated circRNAs expression and 17 down-regulated circRNAs expression. We selected circRNAs which expressed significantly different between radioresistant and radiosensitive cells for further study. Among them, circRNA_101491 has attracted our attention. The circRNA is formed by cyclization of a single exon and comes from the MAPKBP1 gene.

Many studies have shown that circRNA has multiple functions in regulating tumorigenesis and development: (a) circRNA as miRNA sponge [24]; (b) circRNA binding to protein [25]; (c) circRNA translation into protein [26]; (d) transcriptional regulatory factor [27]. we mainly predicted circRNA spongified miRNA so as to affect the radiosensitivity of ESCC in our previous work [23], so this study selected spongified miRNA as the related mechanism of circRNA affecting ESCC radiosensitivity. miRNAs [28, 29] is a large class of short (~ 22nt) ncRNA, which promotes the degradation of target gene mRNA or inhibits its translation by pairing with the 3 ‘UTR (untranslated region) of the target gene. CircRNAs can affect the activity of miRNAs by competing for the binding site of miRNAs. The axial regulation of tumor signal pathway by CircRNA/miRNA is one of the main mechanisms affecting tumor occurrence and development [30]. CircRNAVRK1 inhibits ESCC by regulating the signal pathway of miR-624-3p phosphatase and tensin homologue (PTEN) [31]. We use bioinformatics (RegRNA2.0,http://regrna.mbc.nctu.edu.tw and RNAhybrid, https//bibiserv.cebitec.uni-bielefeld.de/rnahybrid) to predict and filter possible miRNAs candidates. Combined with the expected function of inducing radiation resistance of esophageal cancer cells, four miRNAs (miR-125a-5p; hsa-miR-17-3p hsa-miR-584-5 hsa-miR-562) were selected. miR-125a-5p [33, 34] is a widely expressed miRNAs, which mainly acts as a tumor suppressor gene in various cancers. In addition, it has been reported that miR-125a-5p can inhibit the progress of ESCC [35] and regulate the radiation resistance of LTEP-a2 non-small cell lung cancer cells by targeting sirt7[36]. Through the analysis of bioinformatics software, it was found that there were binding sites between circRNA_101491 and miR-125a-5p. Therefore, whether it is possible for circRNA_101491 to regulate the radiosensitivity of ESCC by regulating miR-125a-5p has become the main aim of our study.

## Materials and methods

### Bioinformatics analysis

Bioinformatics predicts that there are binding sites and interaction potential between TargetScan and miRanda analysis and circRNA101491, and the volcano map of circ expression difference is obtained by circMine online analysis(http://www.biomedical-web.com/circmine/home).

## Cell culture

Purchased from ATCC human esophageal squamous cell carcinoma cell line KYSE-150, RPMI-1640 (10% fetal bovine serum, 100U/mL penicillin, 100 µ g / mL streptomycin) was cultured at 37 ℃ in 5%CO2 incubator.

### Establishment of radiation-resistant cell line KYSE150R

1 × 10^6^ KYSE150 cells suspended in 100 µ L medium were cultured for 2 days, and the linear accelerated 6MV was irradiated with 100 cGy/min dose rate X-rays. After irradiation, the cells were immediately returned to 37 ℃ and 5%CO2 incubator. When the cell growth reaches 90%, trypsin digestion and passage, when the cell proliferation reaches the exponential growth phase, irradiate 1 Gy again, repeat the above steps (1 Gy 3 times, 2 Gy 3 times and 4 Gy 3 times), and gradually establish a radiotherapy-resistant cell line KYSE150R for esophageal cancer, and the cells are cryopreserved in liquid nitrogen. Parental KYSE150 cells were cultured synchronously in the same way during the whole induction process, but without X-ray irradiation.

### Real-time quantitative PCR

Total RNA was extracted by TRIzol, and qPCR analysis was performed by SYBR GreenIMaster with LightCycler ^®^480fluorescence quantitative PCR (Roche). The conditions of PCR are as follows: pre-incubating 5 min at 95 ℃, then amplifying for 10s at 95 ℃, amplifying for 20s at 60 ℃, and finally extending for 15s at 72 ℃. GAPDH or U6 was used as internal reference, and the relative expression of target gene was calculated by 2-delta Ct method. The bulge-loop RT primer and qPCR primers specific for miR-125a-5p and U6 were designed and synthesized by RiboBio, The primer sequences for GAPDH and CircRNA_101491 are shown in Table 1.

**Table 1:** Primer sequence

Genes	primer	Sequences(5’-3’)
GAPDH	Sense	GGTGGTCTCCTGTGACTTCAA
	Antisense	CCACCCTGTTGCTGTAGCC
CircRNA_101491	Sense	GGACAGTACCTTCTGCATCACG
	Antisense	GAACAACACAACCACACACCCT

### Cell transfection

Transient transfection: Negative control (NC), Si-circRNA101491 (5’-TGGGTGGAGCTGAGGGTGT-3’) and miR-125a-5p inhibitor were designed and packed by RiboBio (Guangzhou, China). Cell transfection using Lipofectamine 2000 (Invitrogen, USA).

Stable transfection: Sh-CircRNA10149 in pLenti-U6-shRNA-CMV-GFP-2A-Puro Vector (Human): (5’- CCGGTGGGTGGAGCTGAGGGTGTCTCGAGACACCCTCAGCTCCACCCATTTTTT − 3’) and negative controls (RiboBio China). Cell plank, the cell number should be about 50% on the second day. Cultured overnight at 37 ℃. Before infection, the virus was melted in an ice bath after being removed from the refrigerator at-80 ℃. The original culture medium was absorbed and the virus solution was added to the cell. Shake gently. Cultured overnight at 37 ℃.

### Analysis of radiosensitivity of cells by colony formation assay

ESCC cells of different groups were irradiated with different doses of X-rays (0, 2, 4, 6 and 8GY), and the linear accelerated 6MV was irradiated with 200 cGy/min dose rate X-rays. After cultured at 37 ℃ for one day, the cells in logarithmic phase were digested and inoculated into six-well plate (KYSE150 cells group: 200 cells/well, other groups: 100 cells/well). After normal culture for 14 days, the culture medium was abandoned, 30 min was fixed with 4% paraformaldehyde, and 10 min was stained with crystal violet. After drying, the photos were taken, the clone number was calculated by ImageJ, and use the multi-target click model to analyze survival fraction.

### CCK8 assay

ESCC cells were irradiated with different doses of X-rays (0, 2 and 8 GY), and the linear accelerated 6MV was irradiated with 200 cGy/min dose rate X-rays. inoculated in 96-well plates (1 × 10^3^cells / well). The changes of cell proliferation were detected by CCK8 method 24, 48 and 72 h after the absorbance of each hole was read at the wavelength of 450 nm on the SPARK10M spectrophotometer.

### Transwell assay

The cells of each group were collected and placed in the upper chamber (coated with Matrige). After 24–48 h of culture, the cells migrated to the lower chamber were fixed with methanol and stained with 0.1% crystal violet. Count the number of cells migrated in random observation under an inverted microscope (Olympus).

### Scratch test

Wait until 80% of the cell density converges, and the tip of the sterile pipette is used to artificially form a wound between the cells. PBS washed the cells to grow in the medium containing 2%FBS. The wound closure was observed regularly and photographed under a microscope 12 and 24 h later.

### Western blot

The total protein of each group was taken, and the protein concentration of each group was detected. Protein was separated with 10–12%SDS-PAGE, transferred to PVDF membrane in ice bath for 2 h, 5% skim milk was sealed at room temperature for 2 h, diluted antibody was added and sealed overnight at 4 ℃. Wash the next day, add the corresponding secondary antibody and seal at room temperature for 1 h, then add ECL to obtain the image by film exposure.

### Cell apoptosis, and membrane potential analysis

After 48 h of culture, the treated cells were removed and operated according to the instructions of mitochondrial membrane potential and apoptosis detection kit (Beyotime, China). Mito-Tracker Red CMXRos was red fluorescence, AnnexinV-FITC was green fluorescence and Hoechst33342 was blue fluorescence.

### Animal model of xenografts

Twenty male 4-6-week-old BALB/C-nu/nu nude mice were randomly divided into two groups, and each 0.lml was inoculated subcutaneously on the ventral side of the right flank. The size of the subcutaneous xenografts was measured and recorded every three days. Tumor volume = (length x width^2^)/2. When the volume of the subcutaneous xenografts reached 100mm3, the mice inoculated with KYSE150R + sh- CircRNA_101491 cells were randomly divided into two groups, namely sh- CircRNA_101491 and sh-CircRNA_101491 + IR, with 5 mice in each group. The mice inoculated with NC-KYSE150R cells were randomly divided into two groups, namely NC + IR and NC, with 5 mice in each group. The mice in sh-CircRNA_101491 + IR and NC + IR groups were anesthetized and treated with radiotherapy. The linear accelerated 6MV was irradiated with 200 cGy/min dose rate X-rays. The total dose required for the whole process is 12GY. (6 Gy/ day, a total of 2 days.). After 32 days, the nude mice were killed by anesthesia and the subcutaneous xenografts was isolated. Observe, measure and weigh the subcutaneous xenografts.

### Immunohistochemistry

Dewax and hydrate at room temperature, perform thermal antigen retrieval, replace with a new slice rack, and place the hydrated slices. After antigen retrieval, inactivate endogenous enzyme activity, block non-specific sites, shake off the blocking solution, add primary antibody dropwise to cover the tissue, and then place it in a humidified box at 4 degrees Celsius overnight. (See the antibody instruction manual for primary antibody concentration). The next day, take out the humidified box from the refrigerator at 4 degrees Celsius, add 50 µl of secondary antibody dropwise, and incubate at room temperature for 10 min. Wash with PBS for three minutes three times and distilled water for three minutes. Add one drop or 50 µl of streptozotomycin-peroxidase solution, two drops or 100 µl of DAB solution, and observe under a microscope for 3–10 min. Hematoxylin counterstaining will be terminated after 1–2 min when the cell nuclei turn blue. Decompose with 1% hydrochloric acid alcohol for 3 s and rinse with tap water for three minutes. Dehydration. Take pictures under a microscope.

### Statistical analysis

Use GraphPad Prism 6 to analyze the data, and then display the data as mean ± standard deviation (SD). The statistical significance between different groups was evaluated by double-tailed unpaired t-test between two groups or one-way ANOVA between at least three groups. After statistical treatment, *P* < 0.05 was considered as statistically significant.

## Result

### CircRNA_101491 overexpression in KYSE150R cells

**Fig. 1:**
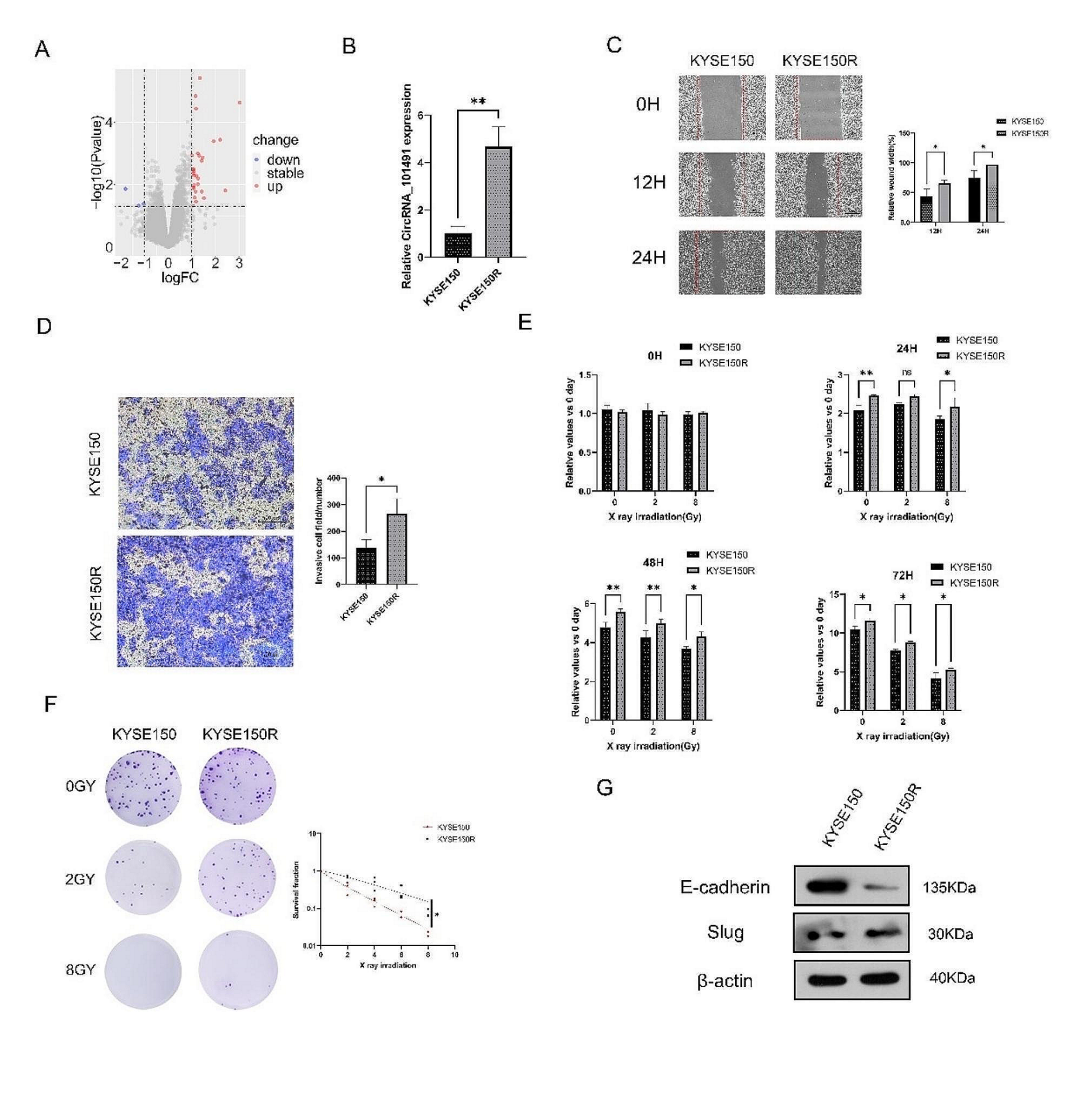
CircRNA_101491 overexpression in KYSE150R cells. (**A**) Volcanic plot of CircRNA_101491 expression. (**B**) Difference in expression level of CircRNA_101491. (C-E) KYSE150R cells have stronger ability to migrate, invade and proliferate. (**F**) colony formation assay showed that the radiosensitivity of KYSE150R cells decreased. (**G**) KYSE150R cells obtained stronger EMT activity. *p < 0.05, **p < 0.01 vs. KYSE150 cells

Bioinformatics analysis and RT-qPCR experiments showed that CircRNA_101491 was overexpressed in KYSE150R (Fig. 1A-B). We further compared the biological activities of the two kinds of cells and found that compared with KYSE150, the migration and invasion ability of KYSE150R was significantly enhanced (Fig. 1C-D). In addition, the proliferation and survival activity of KYSE150R were significantly higher than those of KYSE150 after different doses of X-ray irradiation (Fig. 1E-F). Using the single target multiple hit model, it was found that the radiation resistance of KYSE150R increased significantly (Fig. 1F), which was consistent with our expectations. Moreover, compared with KYSE150, the expression of epithelial marker E-cadherin in KYSE150R cells was down-regulated, while the transcriptional protein slug was significantly increased, indicating that KYSE150R has certain interstitial characteristics (Fig. 1G). The above results suggest that CircRNA_101491 may be involved in the radiation enhancement process of KYSE150R, which is closely related to EMT.

### Silencing CircRNA_101491 affects proliferation, migration, EMT phenotype and radiosensitivity, and accelerates apoptosis

**Fig. 2:**
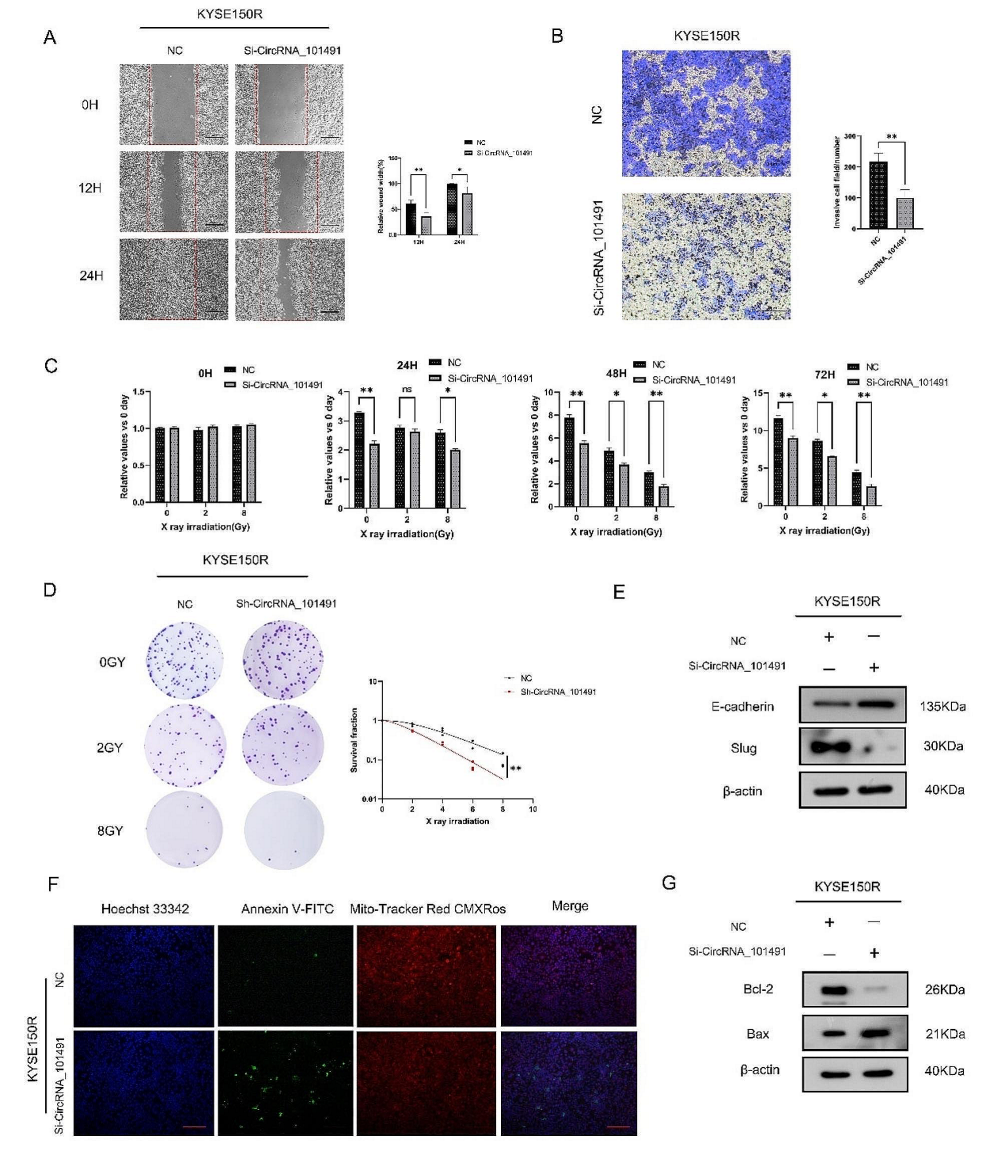
CircRNA_101491 can affect the cell viability and radiosensitivity of KYSE150R. (**A-C**) Silencing the expression of CircRNA_101491 can down-regulate the ability of cell migration, invasion and proliferation. (**D**) Down-regulation of CircRNA_101491 expression can enhance the radiosensitivity of cells. (**E**) Low expression of CircRNA_101491 can reverse EMT activity. (**F-G**) Silencing CircRNA_101491 expression can accelerate the process of apoptosis. *p < 0.05, **p < 0.01 vs. NC

On the basis of the above work, we further studied the role of Circ101491 in regulating radiosensitivity in KYSE150R cells. We silenced CircRNA_101491 in KYSE-150R cells and found that silencing the gene could significantly inhibit the migration, invasion and metastasis of KYSE150R cells (Fig. 2A-B). Further studies showed that after interfering with CircRNA_101491, the viability of KYSE150R cells decreased with the increase of radiation dose. Sh-CircRNA_101491 was transfected into KYSE150R cells by lentiviral vector to stably block the expression of CircRNA_101491 (Fig. 2C). We found that the clone number of silenced KYSE150R cells decreased significantly under the irradiation doses of 0GY, 2GY and 8GY, which confirmed that the viability of silenced KYSE150R cells decreased. We also used the multi-target click model to analyze the survival score of silenced KYSE150R cells (Fig. 2D). The results showed that the survival score of silenced KYSE150R cells was significantly higher than that of the control group. It is not difficult to see that silenced CircRNA_101491 significantly improved the radiosensitivity of KYSE150R cells. Epithelial mesenchymal transformation (EMT) [37, 38] is a process mainly related to radiation resistance of cancer. Therefore, EMT may be an effective way to treat radiation resistance of cancer. We found that after silencing CircRNA_101491, the expression of epithelial marker protein E-cadherin increased and the expression of transcriptional protein Slug decreased (Fig. 2E). Apoptosis was detected by green fluorescent probe AnnexinV-FITC and mitochondrial membrane potential was measured by red fluorescent probe Mito-Tracker Red CMXRos. We found that after knockout CircRNA_101491 expression in KYSE150R cells, the green fluorescent cells increased significantly, suggesting that the cell activity of knockout CircRNA_101491 was inhibited. On this basis, more red light was observed in the cells of the NC group, which indicated that the membrane potential of the cells was stable in the normal control group (Fig. 2F). Further studies showed that interfering with CircRNA_101491 could up-regulate the expression of Bax and down-regulate the expression of Bcl-2 (Fig. 2G). indicating that silencing CircRNA_101491 inhibits the acquisition of interstitial properties of KYSE150R cells, which reduces the proliferation, migration and radiation resistance of esophageal cancer cells, and accelerates the process of apoptosis.

### CircRNA_101491 competitive binding with miR-125a-5p regulates the radiosensitivity of KYSE150R cells

**Fig. 3:**
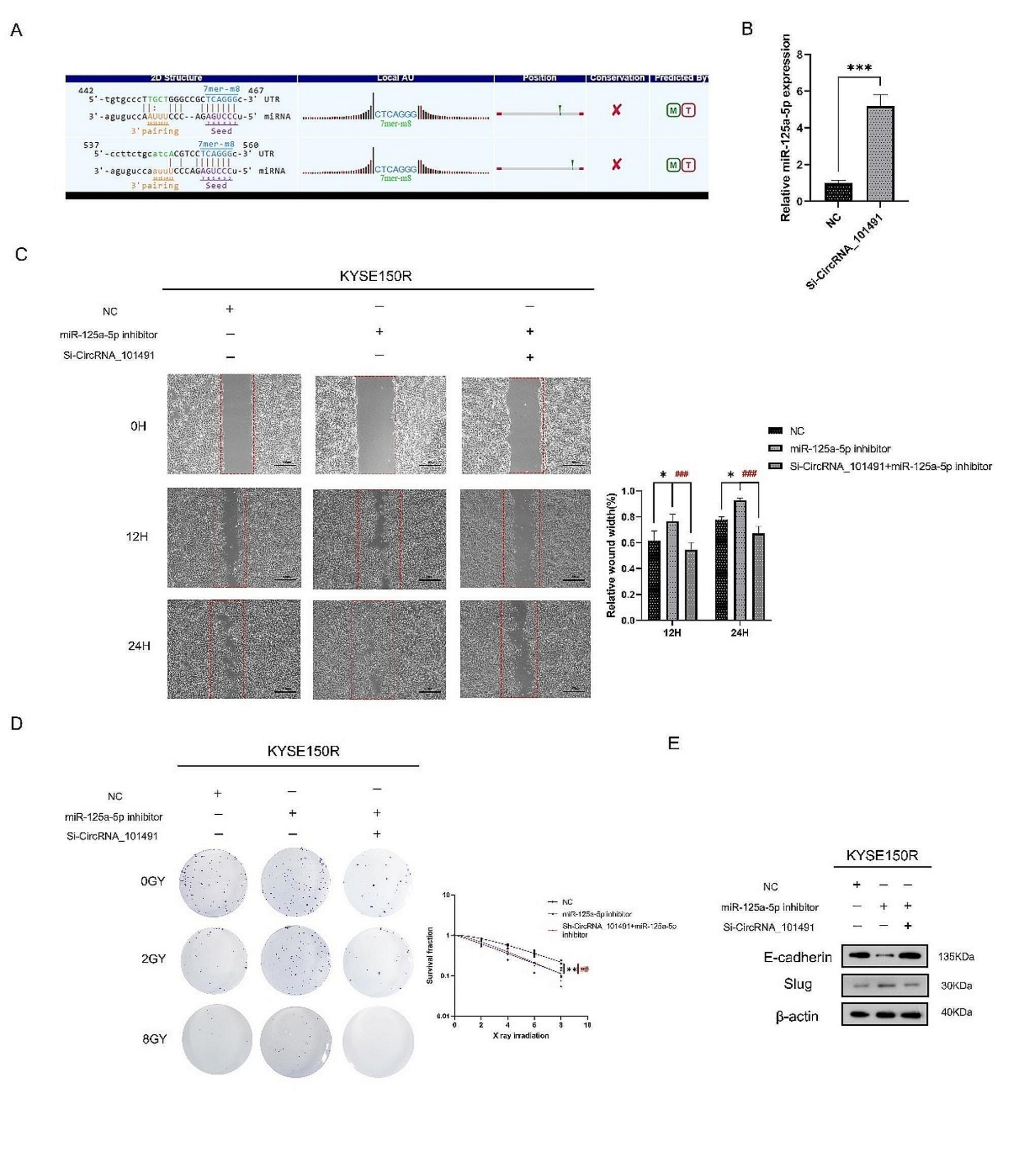
CircRNA_101491 exerts its ability mainly by combining with miR-125a-5p. (**A**) Binding sites between CircRNA_101491 and miR-125a-5p. (**B**) After inhibiting the expression of CircRNA_101491, the expression of miR-125a-5p was significantly increased. (**C**-**D**) Inhibition of miR-125a-5p expression can accelerate cell migration and reduce radiosensitivity, while decreasing CircRNA_101491 expression can reverse this process. (**E**) Western blot revealed that inhibition of miR-125a-5p expression could accelerate the transformation of cells to stroma, but knocking down the expression of CircRNA_101491 could restore the phenotype of epithelial cells. *p < 0.05, **p < 0.01, ***p < 0.001 vs. NC; ^##^*p* < 0.01, ^###^*p* < 0.001 vs. miR-125a-5p inhibitors

As shown in Fig. 3.A, Bioinformatics predicts the binding sites and binding forms of miR-125a-5p and CircRNA_101491. To further prove that miR-125a-5p is the target of CircRNA_101491, we detected the expression of miR-125a-5p in KYSE150R cells transfected with Si-CircRNA_101491 by RT-qPCR. The results showed that compared with NC group, the expression of miR-125a-5p in Si-CircRNA_101491 group was significantly up-regulated (Fig. 3.B). Therefore, we speculate that CircRNA_101491 does inhibit the expression of miR-125a-5p. MiR-125a-5p inhibitors accelerated the migration of KYSE150R cells, while co-transfection with Si-CircRNA_101491 significantly slowed down the migration of KYSE150R cells (Fig. 3.C). In addition, miR-125a-5p inhibitors significantly increased the number of clones in 0GY, 2GY and 8GY, but after co-transfection with Si-CircRNA_101491, the number of clones decreased significantly. Through the analysis of survival fraction by multi-target click model, it was observed that miR-125a-5p inhibitor could increase the radiation resistance of KYSE150R cells, while co-transfection of Si-CircRNA_101491 could reverse this process (Fig. 3.D). Moreover, we found that the expression of EMT-related protein E-cadherin was decreased and Slug was up-regulated after transfection of miR-125a-5p inhibitors (Fig. 3.E), which proved that miR-125a-5p inhibitors could accelerate the EMT progression of KYSE150R cells, while co-transfection of Si-CircRNA_101491 could inhibit the progression of EMT. This suggests that miR-125a-5p does have the ability to inhibit cell migration and EMT progression, and further reduce the radiation resistance of cells, which is inhibited by CircRNA_101491. Therefore, we can conclude that CircRNA_101491 controls the progress of EMT by competitively binding to miR-125a-5p, and then regulates the radiation resistance of KYSE150.

### Silencing CircRNA_101491 can increase the radiosensitivity of KYSE150R cells xenografts

**Fig. 4:**
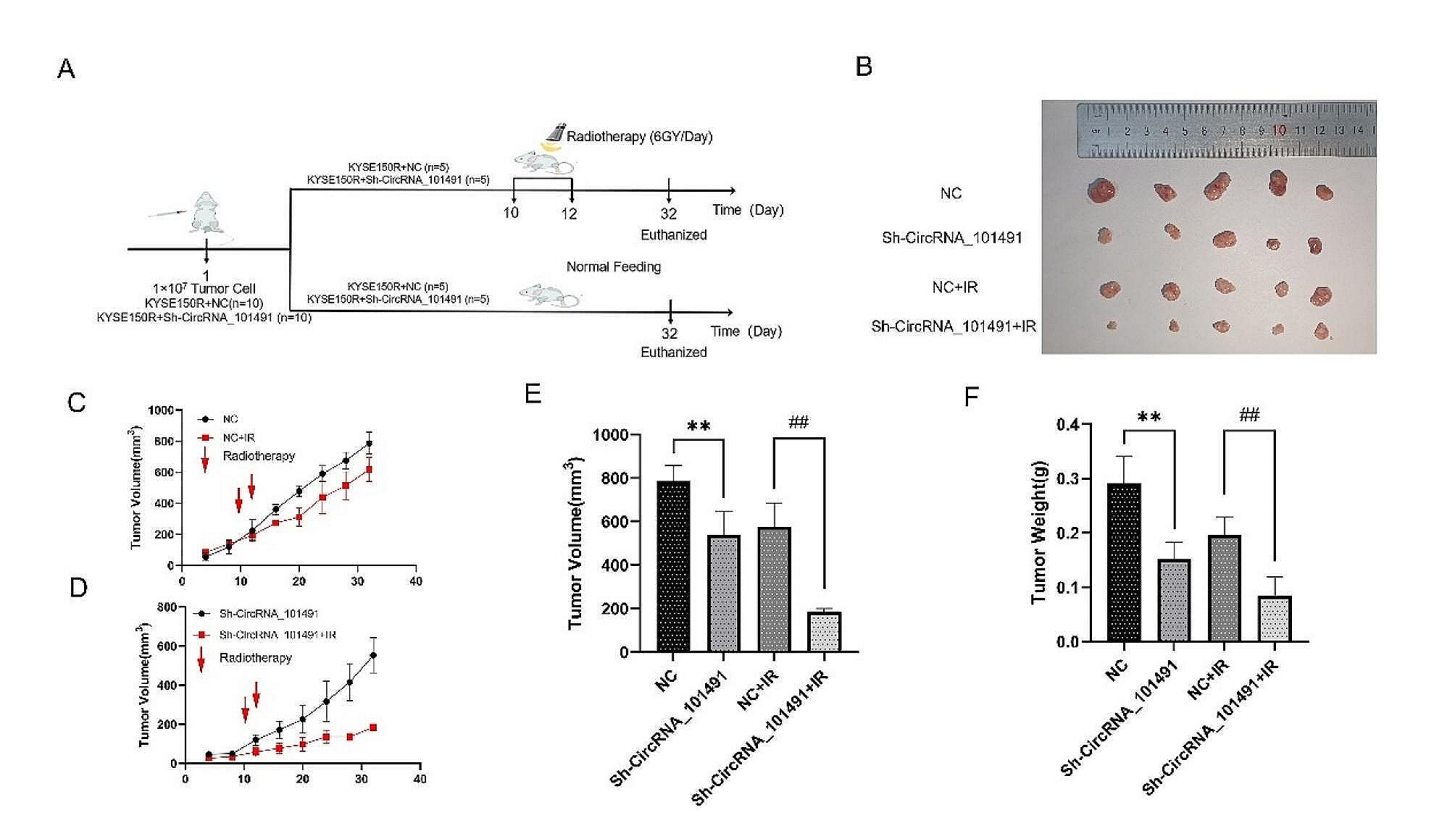
Effect of CircRNA_101491 on tumor tissue in vivo (**A**) Flow chart of in vivo experiment. (**B**) Naked eye map of tumor tissue. (**C-D**) The growth rate of tumor tissue in each group under different treatments. (**E-F**) Changes of tumor tissue volume and weight in each group after different treatments. **p < 0.01 vs. NC; ^##^*p* < 0.01 vs. NC + IR

According to the results of in vitro experiments, we preliminarily identified that silencing CircRNA_101491 could enhance the radiosensitivity of KYSE150R cells, so we wanted to further verify the role of CircRNA_101491 in vivo, so we selected nude mice and injected different groups (NC group; Sh-CircRNA_101491 group; NC + IR group; Sh-CircRNA_101491 + IR group) of tumor cells subcutaneously into nude mice to form a subcutaneous xenografts model. On the 10th and 12th day, the subcutaneous tumor was locally irradiated, the linear accelerated 6MV was irradiated with 200 cGy/min dose rate X-rays. the total dose required for the whole process was 12GY. (6GY/ day, a total of 2 days. Figure 4A). The volume of the tumor was measured every 4 days. After 32 days, the mice were euthanized and the tumor was removed (Fig. 4B). Results We found that after IR treatment, due to the radiation resistance of KYSE150R cells, the effect of radiotherapy on tumor growth rate is not significant (Fig. 3C), but after silencing the expression of CircRNA_101491, we could see that radiotherapy can significantly slow down tumor growth rate (Fig. 3D). This suggests that the silent expression of CircRNA_101491 can increase the radiosensitivity of KYSE150R cells xenografts. The tumor volume and weight in Sh-CircRNA_101491 + IR group were the smallest (Fig. 4E-F), meanwhile, we calculated the tumor reduction rate according to (average volume of unirradiated tumor-average maximum volume of tumor after radiotherapy)/average maximum volume of unirradiated tumor, and finally concluded that the volume and weight reduction rate of Sh-CircRNA_101491 + IR group was the highest (Table 2). The above results suggest that silencing CircRNA_101491 can enhance the radiosensitivity of KYSE150R cells xenografts.

**Table 2:** Tumor reduction rate after different treatments

Group	Tumor volume (mm3)	Tumor weight (g)	Volume inhibition (%)	Weight inhibition (%)
NC	785.86 ± 60.85	0.29 ± 0.42	/	/
Sh-CircRNA_101491	540.28 ± 94.13	0.15 ± 0.27	/	/
NC + IR **(vs. with NC)**	578.31 ± 93.38	0.20 ± 0.029	26.41%	32.88%*
Sh-CircRNA_101491 + IR **(vs. with Sh-CircRNA_101491)**	185.24 ± 13.90	0.08 ± 0.03	65.71%^##^	43.90%^##^

*p < 0.05 vs. NC; ^##^*p* < 0.01 vs. NC + IR

### Molecular mechanism of CircRNA_101491 regulating radiosensitivity of xenografts in vivo

**Fig. 5:**
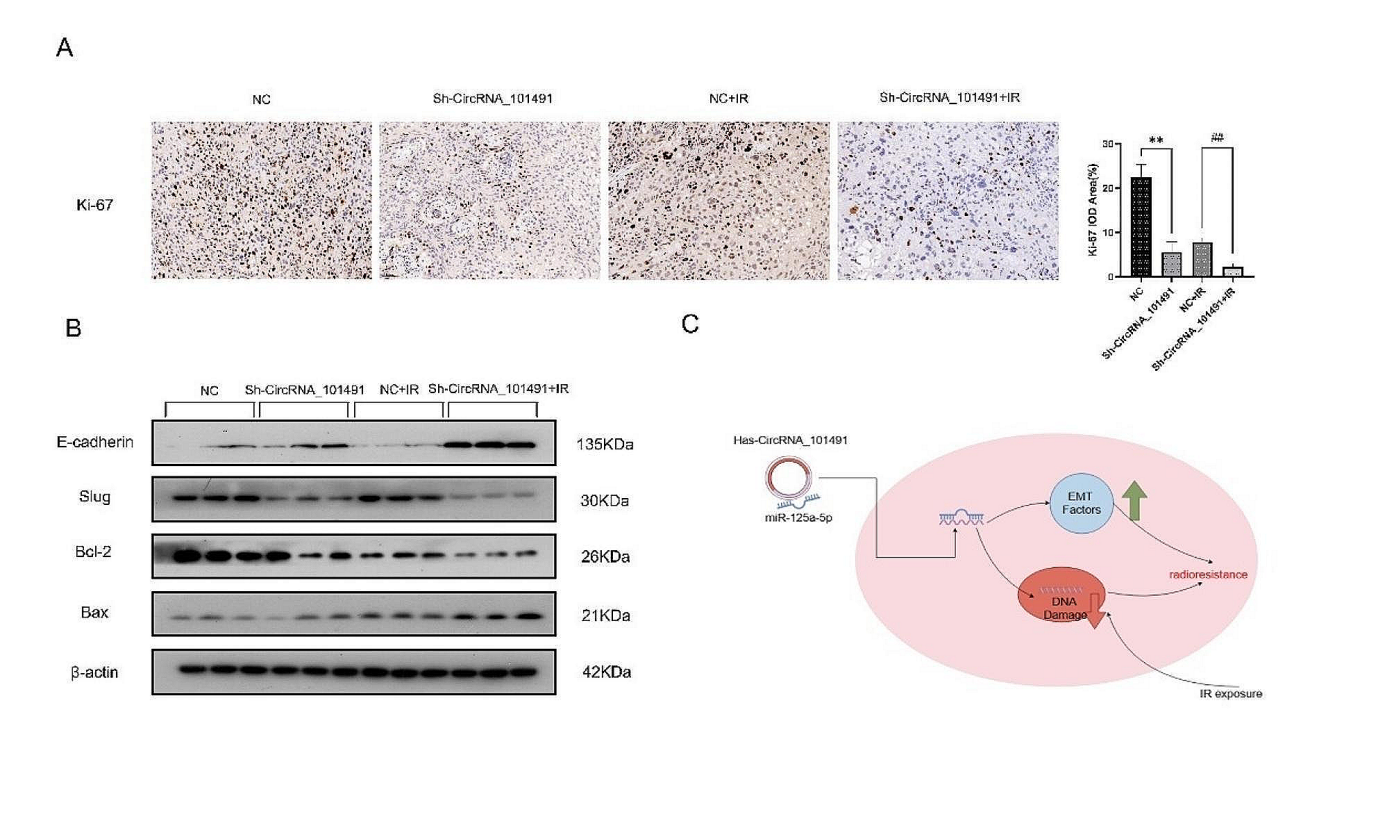
The related mechanism of the effect of CircRNA_101491 on tumor tissue in vivo (**A**) Expression of Ki-67 in tumor tissues of each group. (**B**) Expression of EMT-related proteins and apoptotic family-related proteins in tumor tissues of each group. (**C**) The mechanism diagram of this study. (IR: Infrared Radiation) **p < 0.01 vs. NC; ^##^*p* < 0.01 vs. NC + IR

Ki-67[39] has always been regarded as a marker of tumor cell proliferation. Therefore, we use immunohistochemical experiments to verify the expression of Ki-67 in each group of tumor tissues, and found that the expression of Ki-67 in Sh-CircRNA_101491 + IR group is the lowest (Fig. 5.A), which means that silencing CircRNA_101491 can increase the radiosensitivity of xenografts. At the same time, we found that the E-cadherin expression of EMT protein in Sh-CircRNA_101491 + IR group was the highest, while the expression of Slug was the lowest (Fig. 5.B). Therefore, it can be concluded that CircRNA_101491 regulates the radiosensitivity of xenografts by controlling the intracellular EMT process. Moreover, we also found that silencing CircRNA_101491 can accelerate the process of apoptosis of xenografts, which is consistent with the results of our in vitro experiment.

## Discussion

Circular RNA (CircRNAs) [13, 40, 41], which is highly stable and rich in content, is a kind of important tumor markers and can be used in tumor radiotherapy and chemotherapy. Radiotherapy is a very important treatment in clinic at present, but the therapeutic effect is not good because many tumor cells are not sensitive to X-rays [42]. The acquired radiotherapy resistance of tumor cells is a major problem in the treatment of many cancers. How to overcome their resistance to radiotherapy is an urgent clinical problem to be solved.

In recent years, more and more studies [43–45] have shown that circRNA plays an important role in regulating the radiosensitivity of ESCC. In our study, we first constructed KYSE150R cells and successfully detected that the response of KYSE150R cells to radiotherapy was limited. At the same time, it was also found that the expression of CircRNA_101491 in KYSE150R was significantly higher than that in KYSE150, so to explore the specific role of CircRNA_101491 in KYSE150R, we introduced Si-CircRNA_101491/Sh-CircRNA_101491 into KYSE150R to observe the effect of CircRNA_101491 on radiosensitivity of esophageal squamous cell carcinoma. The results showed that after transfection, the migration, invasion and proliferation activity of KYSE150R cells were significantly decreased, while the radiosensitivity was significantly increased. These results suggest that silencing the expression of CircRNA_101491 can reduce the viability of KYSE150R cells and increase the radiosensitivity.

CircRNAs generally exerts its biological activity by targeting miRNA [46]. Therefore, in this study, bioinformatics method was used to screen out the binding site between CircRNA_101491 and miR-125a-5p, and then RT-qPCR quantitative analysis confirmed that the expression of miR-125a-5p was significantly increased after transfection of Si-CircRNA_101491, which suggested that miR-125a-5p was indeed the target gene of CircRNA_101491 and had an inhibitory effect on miR-125a-5p. On the basis of this work, we further explore the radiosensitivity and phenotypic mechanism of miR-125a-5p to esophageal cancer cells. The results show that miR-125a-5p inhibitors can increase cell migration and reduce the radiosensitivity of KYSE150R cells, while the addition of Si-CircRNA_101491 co-transfection can reverse this process. Therefore, we can conclude that CircRNA_101491 may reduce the radiosensitivity of esophageal cancer cells by competitively inhibiting the expression of miR-125a-5p.

Epithelial-mesenchymal transformation (EMT) [10] is a process in which epithelial cells have interstitial characteristics (such as migration, invasion, etc.) in a short period of time, which is the key link of tumor invasion, metastasis and radiation resistance. Therefore, in this study, through Western blotting, we found that KYSE150R cells have higher EMT ability than parent cell KYSE150. This suggests that EMT is indeed involved in the process of KYSE150R cells acquired radiotherapy resistance. After that, we explored whether the CircRNA_101491/miR-125a-5p axis reduced the radiosensitivity of esophageal cancer by inhibiting EMT. The results showed that the transfection of miR-125a-5p inhibitor did enhance the EMT ability of esophageal cancer cells, and then we knocked down CircRNA_101491 on the basis of transfection of miR-125a-5p inhibitor, and found that the up-regulation of EMT ability was reversed. The above experiments show that CircRNA_101491/miR-125a-5p can affect the radiosensitivity of esophageal cancer cells by affecting the EMT process of cells.

## Conclusion

To sum up, the high expression of CircRNA_101491 is related to the radiosensitivity of ESCC. Silencing CircRNA_101491 can reduce the proliferation and migration of KYSE150R cells in vitro and enhance the radiosensitivity. In addition, miR-125a-5p has been proved to be the downstream target of CircRNA_101491 to regulate the radiosensitivity of ESCC, and EMT is also a key link in the regulation of ESCC radiosensitivity by CircRNA_101491/miR-125a-5p axis. Therefore, this study will provide a potential target for reducing radiotherapy failure in patients with esophageal cancer.

### Electronic supplementary material

Below is the link to the electronic supplementary material.

Supplementary Material 1

## Data Availability

Data is provided within the manuscript or supplementary information files.
