# Supplementary figures and images for: CircRNA_101491 regulated the radiation sensitivity of esophageal squamous cell carcinomas via sponging miR-125a-5p
Source: Radiat Oncol. 2024 Jun 26;19:84. doi: 10.1186/s13014-024-02478-7 (PMC11210101; doi:10.1186/s13014-024-02478-7)

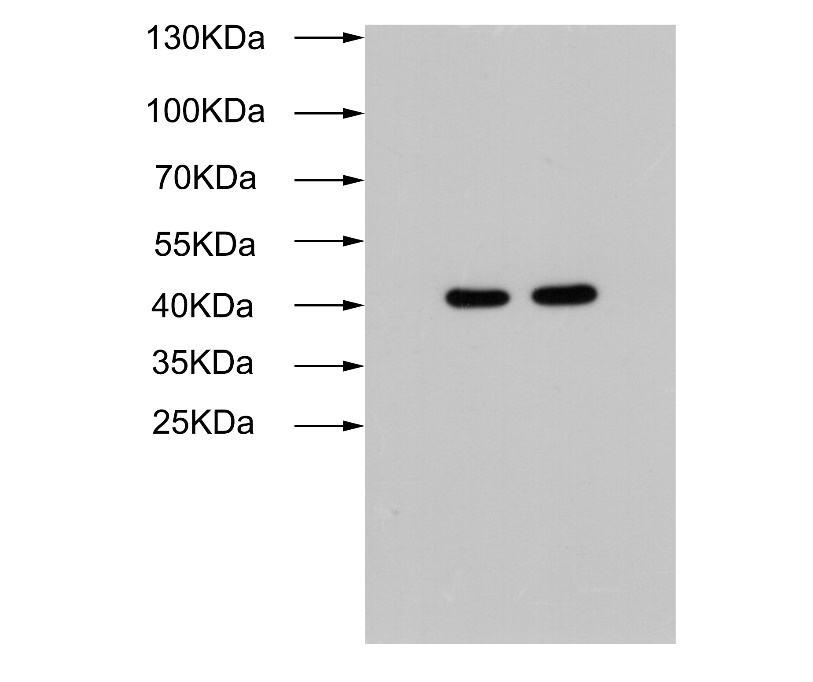

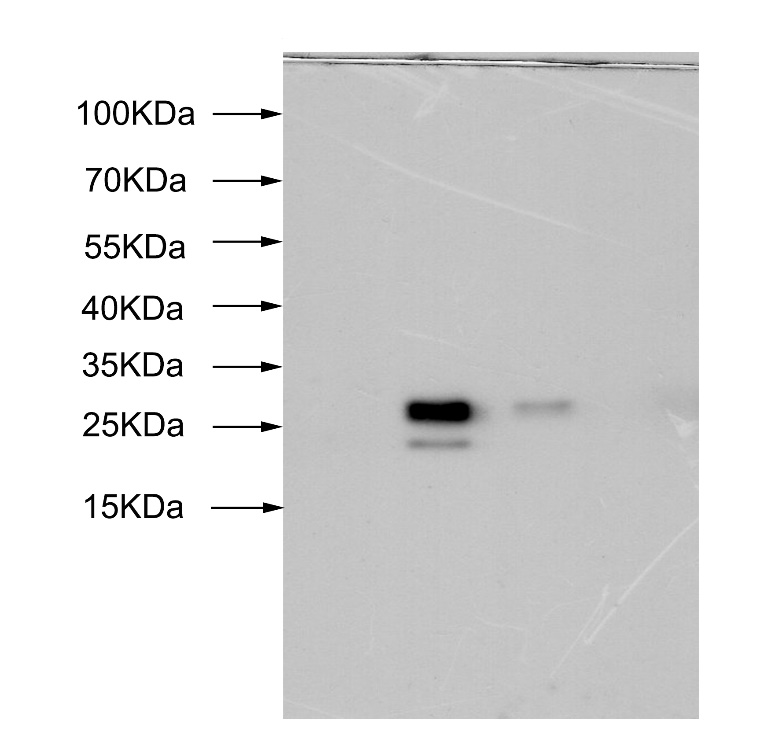

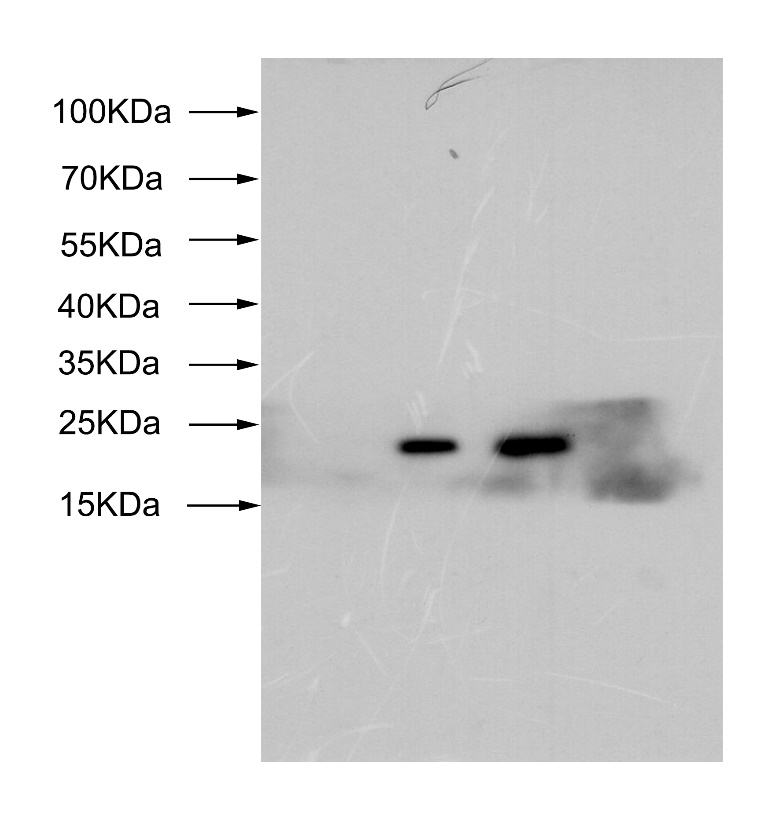

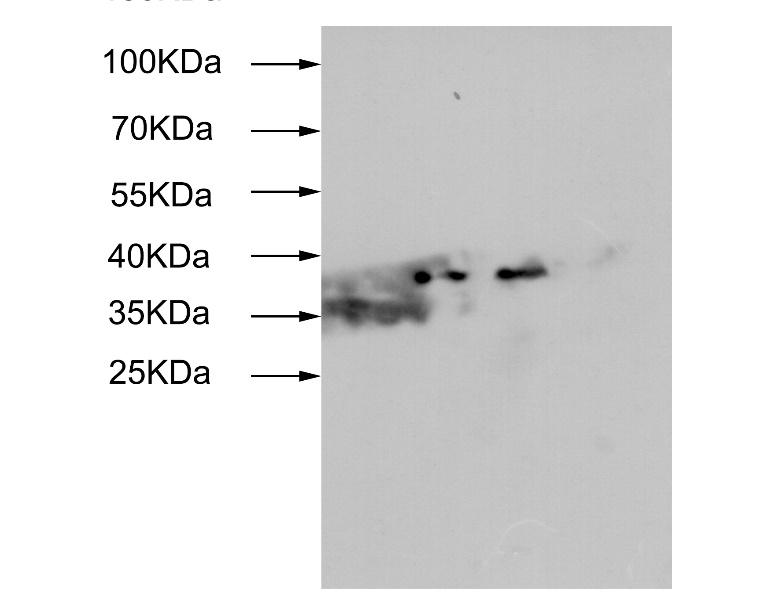

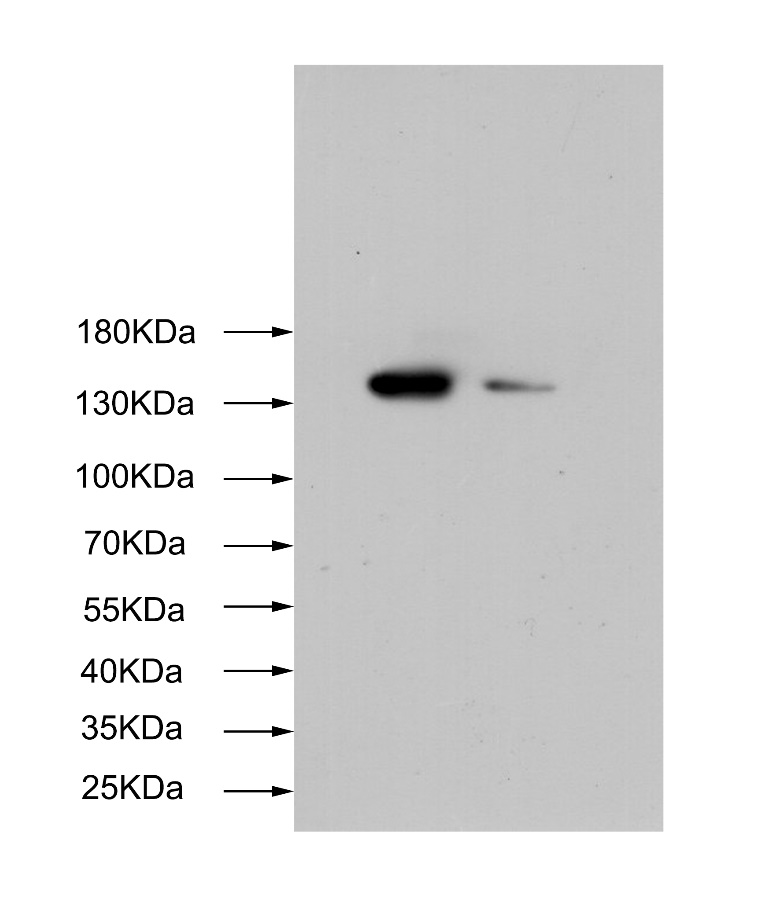

Supplement: Supplementary file 1 — Supplementary Material 1 [file 13014_2024_2478_MOESM1_ESM.docx]
